# Comparative efficacy and acceptability of five anti-tubercular drugs in treatment of multidrug resistant tuberculosis: a network meta-analysis

**DOI:** 10.1186/s13336-015-0020-x

**Published:** 2015-04-28

**Authors:** Huaidong Wang, Xiaotian Zhang, Yuanxiang Bai, Zipeng Duan, Yan Lin, Guoqing Wang, Fan Li

**Affiliations:** Key Laboratory of Zoonosis, Ministry of Education, College of Basic Medicine, Jilin University, 126 Xinmin Street, Changchun, Jilin China

**Keywords:** Network meta-analysis, Antitubercular drugs, Multidrug-resistant tuberculosis

## Abstract

Multidrug resistant tuberculosis (MDR-TB) is a serious form of tuberculosis (TB). There is no recognized effective treatment for MDR-TB, although there are a number of publications that have reported positive results for MDR-TB. We performed a network meta-analysis to assess the efficacy and acceptability of potential antitubercular drugs. We conducted a network meta-analysis of randomized controlled clinical trials to compare the efficacy and acceptability of five antitubercular drugs, bedaquiline, delamanid, levofloxacin, metronidazole and moxifloxacin in the treatment of MDR-TB. We included eleven suitable trials from nine journal articles and six clinical trials from ClinicalTrials.gov, with data for 1472 participants. Bedaquiline (odds ratio [OR] 2.69, 95% CI 1.02-7.43), delamanid (OR 2.45, 95% CI 1.36-4.89) and moxifloxacin (OR 2.47, 95% CI 1.01, 7.31) were significantly more effective than placebo. For efficacy, the results indicated no statistical significance between each antitubercular drug. For acceptability, the results indicated no statistically significant difference between each compared intervention. There is insufficient evidence to suggest that any one of the five antitubercular drugs (bedaquiline, delamanid, levofloxacin, metronidazole and moxifloxacin) has superior efficacy compared to the others.

**Electronic supplementary material:**

The online version of this article (doi:10.1186/s13336-015-0020-x) contains supplementary material, which is available to authorized users.

## Introduction

Tuberculosis (TB) has tormented humans over the centuries and was a leading cause of death in Asia, Europe and North America for centuries [1,2]. The emergence of multidrug resistance tuberculosis (MDR-TB) and even extensively drug tuberculosis resistant (XDR-TB) is a major threat to global TB care and control. MDR-TB is caused by *Mycobacterium tuberculosis* that is resistant to at least isoniazid (INH) and rifampin (RMP). MDR-TB includes the subcategory of XDR-TB, which is MDR-TB with additional resistance to any fluoroquinolone and to at least one of three injectable anti-TB drugs [3]. The World Health Organization (WHO) estimates that about 450,000 new MDR-TB cases and about 170,000 MDR-TB deaths have occurred across the world in 2012 [4].

Currently, MDR-TB treatments are not satisfactory, because of the long treatment cycle and high cost. The overall treatment duration is at least 18 months, and requires more toxic and less effective agents than that used for drug-susceptible TB [5]. Only 48% of patients with MDR-TB have been successfully treated in 2010, with more patients (52%) has been withdrawn as a reason of adverse effects or deaths according to the World Health Organization (WHO). Currently, second-line and third-line agents such as ofloxacin, levofloxacin, moxifloxacin and linezolid have been used for the treatment of MDR-TB and XDR-TB, owing to the drug resistance of first-line antituberculosis drugs [6]. However, some of these drugs are not recommended for routine use due to variable efficacy and serious side effects. Therefore, it is necessary to develop effective drugs for MDR-TB treatment. Some new anti-MDR-TB drugs are currently at various stages of clinical evaluation or preclinical development such as bedaquiline (TMC207) and delamanid (OPC-67683) [7]. Bedaquiline was registered by the FDA of the United States of America in December 2012 and has been recommended for use in adults with MDR pulmonary tuberculosis by WHO [8]. Delamanid is awaiting approval by FDA and Pharmaceutical and Medical Devices Agency (PMDA) of Japan [9]. In addition, existing drugs such as levofloxacin and moxifloxacin are also being applied to the treatment of MDR-TB (Additional file 1: Table S1) [10-12]. Some new drugs that are at an earlier stage in the drug development pipeline such as PA-824 and SQ109 also have shown promise. In the face of emerging new anti-TB treatments, an important question is regarding the assessment of the efficacy and acceptability of these drugs, and establishment of optimal treatment regimes.

The evidence derived from multiple trials and cohort studies on MDR-TB treatment has been a subject of much debate and has not provided useful guidance to clinicians. Traditional meta-analyses were unable to provide comprehensive comparisons between individual anti MDR-TB drugs because they failed to integrate all available randomized evidence within one analysis. Network meta-analysis is a type of meta-analysis in which, rather than simply summing up trials that have evaluated the same treatment compared to placebo, different treatments are compared by statistical inference. We can use network meta-analysis for assessing the relative efficacy and acceptability of new antitubercular MDR-TB drugs, especially where there is a shortage of pair-wise comparison of direct clinical trials of two antitubercular drugs.

We aimed to compare the five antitubercular drugs, bedaquiline, delamanid, levofloxacin, metronidazole, moxifloxacin in the treatment of MDR-TB. Our intention was to analyze the efficacy and acceptability of antitubercular MDR-TB drugs by integrating all available direct and indirect evidence using network meta-analyses.

## Methods

### Study selection and data collection

We did a network meta-analysis to compare five antitubercular drugs for MDR-TB. We included all randomized trials comparing one antitubercular drug at a therapeutic dose with other antitubercular drugs or with placebo as oral therapy for adults with MDR-TB before October 8, 2014. The participants were both men and women, aged 18 years or older, and with a primary diagnosis of tuberculosis according to standard diagnostic criteria. The patients who had sputum culture–positive multidrug-resistant tuberculosis, with confirmed genotypic or phenotypic resistance to isoniazid, rifampin, and chest radiographic findings consistent with tuberculosis were diagnosed with MDR TB. Both fixed-dose and flexible-dose designs were allowed. We excluded trials that assess early bactericidal activity and pharmacokinetics.

We searched Embase (http://www.elsevier.com/online-tools/embase), Pubmed (http://www.ncbi.nlm.nih.gov/pubmed/), ClinicalTrials.gov (http://www.clinicaltrials.gov/), the US Food and Drug Administration website (http://www.fda.gov/) and CNKI (http://www.cnki.net/), using the following keywords: “multidrug resistance tuberculosis, drug resistance tuberculosis, MDR-TB clinical, DR-TB clinical, randomized clinical of MDR-TB, MDR-TB treatment”. Additionally, we reviewed the meta-analyses and publications for other potential data sources related to antitubercular MDR-TB drugs. Study participants were required to have a clinical diagnosis of MDR-TB. Data extraction was performed independently by two of the authors and checked by another. To assess the methodological quality of included trials we used the criteria for quality assessment recommended by the Cochrane risk-of-bias [13], which takes into account methodological errors arising from aspects such as allocation concealment, blinding, completeness of outcome data, selective outcome reporting and other potential sources of bias.

### Outcome measures

Response and dropout rates were chosen as primary outcomes reported estimates of treatment efficacy and acceptability. We defined response as the number of patients whom showed sputum culture conversion to negative. We defined treatment acceptability as the number of patients who completed the therapy.

### Statistical analysis

The whole meta-analysis in this study was divided into two sections, including direct comparison from traditional meta-analysis and indirect comparison from network meta-analysis. We performed a traditional meta-analysis to yield the Mantel-Haenszel odds ratio firstly. If a trial result was presented with zero events in one group, then the event rate was artificially inflated by adding 0.5; if a trial result was presented with zero in both groups, then the data was excluded. Then, we performed traditional meta-analysis used Stata version 12.0 to analyze the heterogeneity of each study. Heterogeneity between trials was quantified with the I^2^ and H measure. If heterogeneity was moderate or great, we performed meta-analysis by comparing the same interventions with a random-effects model [14]. According to the heterogeneity, we choose a random-effects model to do the following analysis.

In addition, we performed network meta-analysis using random-effects model in R2WinBUGS [15] in R operating environment. We modeled the binary outcomes in every treatment group of every study, and specified the relations among the odds ratios (ORs) across studies to make different comparisons [16]. This method combines direct and indirect comparison for any given pair of treatments. Analyses were performed in the statistical package R 3.0.2. Finally, we analyze the fitting results and consistency of network meta-analysis.

We also examined the pairwise comparative of efficacy and acceptability among the five antitubercular drugs. We expressed the results using bedaquiline as reference drugs, because bedaquiline was the first new tuberculosis drug approved by Food and Drug Administration (FDA) for MDR-TB patients [17]. This method is an indirect comparison, and we performed this meta-analysis using the one-line program published by Lumley [18].

## Results

### Included trials

**Figure 1:**
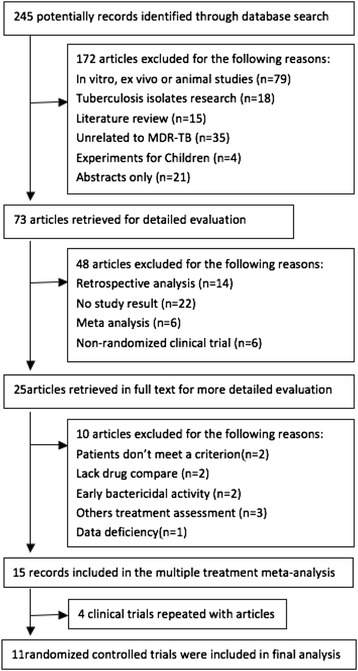
Flow diagram of the study.

**Figure 2:**
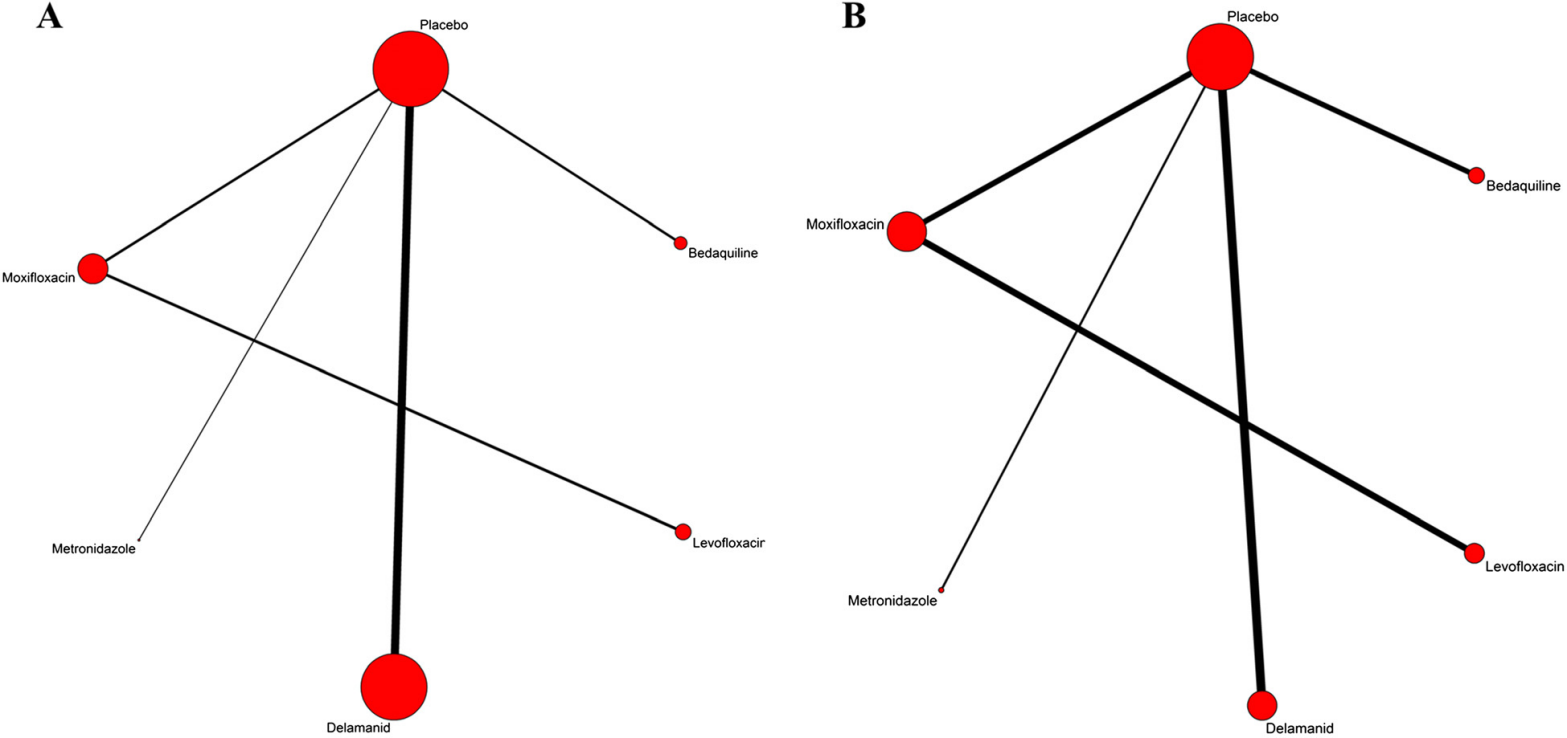
Network meta-analysis of eligible clinical trials of antitubercular drugs for MDR-TB. **A**: Eligible clinical trials of efficacy; **B**: Eligible clinical trials of acceptability. The lines represent direct comparison trials, and the width of the lines is proportional to the number of trials comparing each pair of treatments, and the size of each node is proportional to the number of randomised participants (sample size).

The electronic searches yielded 245 potentially relevant studies, of which 73 potentially eligible articles were analyzed. We excluded 48 reports that did not meet eligibility criteria (Figure 1). Overall, we used eleven trials from 2009 to 2013 for the network meta-analysis (including 9 journal articles and 6 clinical trials). Detailed characteristics of all studies included in the meta-analysis are listed in Table 1 and Table 2. The methodological quality of included trials was generally high (the risk of bias of these clinical trials shown in Additional file 2: Figure S1). Delamanid was evaluated in the maximum number of patients (711) whereas metronidazole was evaluated in the least number (70). Moxifloxacin was evaluated in the maximum number of trials (4). Bedaquiline and delamanid had the largest number of patient follow-ups (207 and 711 overall, respectively), whereas moxifloxacin and levofloxacin had no follow-ups described. Overall, 1472 individuals were randomly assigned to one of the five antitubercular agents and were included in the network meta-analysis. Figure 2 shows the network of eligible comparisons for the network meta-analysis. 1242 individuals were included in the efficacy analysis (10 trials) and 1244 in the acceptability analysis (9 trials). Most of placebo treatment used background drug regimen treatment plus placebo, others were not in detail. Regional disparity also exists in treatment in background drug regimen treatments. The mean duration of the studies was 21.1 weeks and the mean sample size was 134 participants per group (range 35-481). Most trials were carried out in Korea (Table 2).

**Table 1:** **Characteristics of included trials**

**Trial**	**Interventions**	**Dose (mg/day)**	**Comparator**	**No of patients**	**Course (weeks)**	**Follow-up (weeks)**	**Source**
DiaconAH 2009,2012	Bedaquiline	1600,1200	placebo	47	8	104	journal article [22,23]
Trail NCT00449644	Bedaquiline	1600,1200	placebo	160	24	104	clinical trial website [24], study register
Gler MT 2012	Delamanid	400,200	placebo	481	8	104	journal article [25], clinical trial website [26]
Trial NCT00685360	Delamanid	400,200	placebo	192	8	104	journal article [27],
Zhang Q 2013	Delamanid	400,200	placebo	38	24	104	journal article [28]
Carroll MW 2013	Metronidazole	1500	placebo	35	8	24	journal article [29]
Trail NCT00425113	Metronidazole	1500	placebo	35	8	72	clinical trial website [30]
Trail NCT00082173	Moxifloxacin	400	placebo	146	8	0	clinical trial website [31]
Chen Y 2013	Moxifloxacin	400	placebo	74	52	0	journal article [32]
Koh WJ 2013	Moxifloxacin	400	levofloxacin	90	12	0	journal article [33], clinical trial website [34]
Liang LL 2011	Moxifloxacin	400	levofloxacin	46	72	0	journal article [35]
Koh WJ 2013	Levofloxacin	750	moxifloxacin	92	12	0	journal article [33], clinical trial website [34]
Liang LL 2011	Levofloxacin	600	moxifloxacin	36	72	0	journal article [35]

BR: background drug regimen treatment, INH: isoniazid, PZA: pyrazinamide, MOX: moxifloxacin, EMB: ethambutol, RIF: rifampicin, OFL: ofloxacin, AMK: amikacin.

**Table 2:** **Studies included in the network meta-analysis**

**Interventions**	**No of trials**	**Range(mg/day)**	**Year of publication**	**Country**
Bedaquiline	2	1200-1600	2009-2012	Brazil, India, Latvia, Peru, Russian Federation, South Africa, Thailand
Delamanid	3	200-400	2012-2013	China, Egypt, Estonia, Japan, Korea, Latvia, Peru, Philippines, Cairo, Masan, United States.
Metronidazole	2	1500	2013	Korea
Moxifloxacin	4	400	2011-2013	Brazil, Korea,China
Levofloxacin	2	600-750	2011-2013	Korea, China

### Direct comparisons

**Figure 3:**
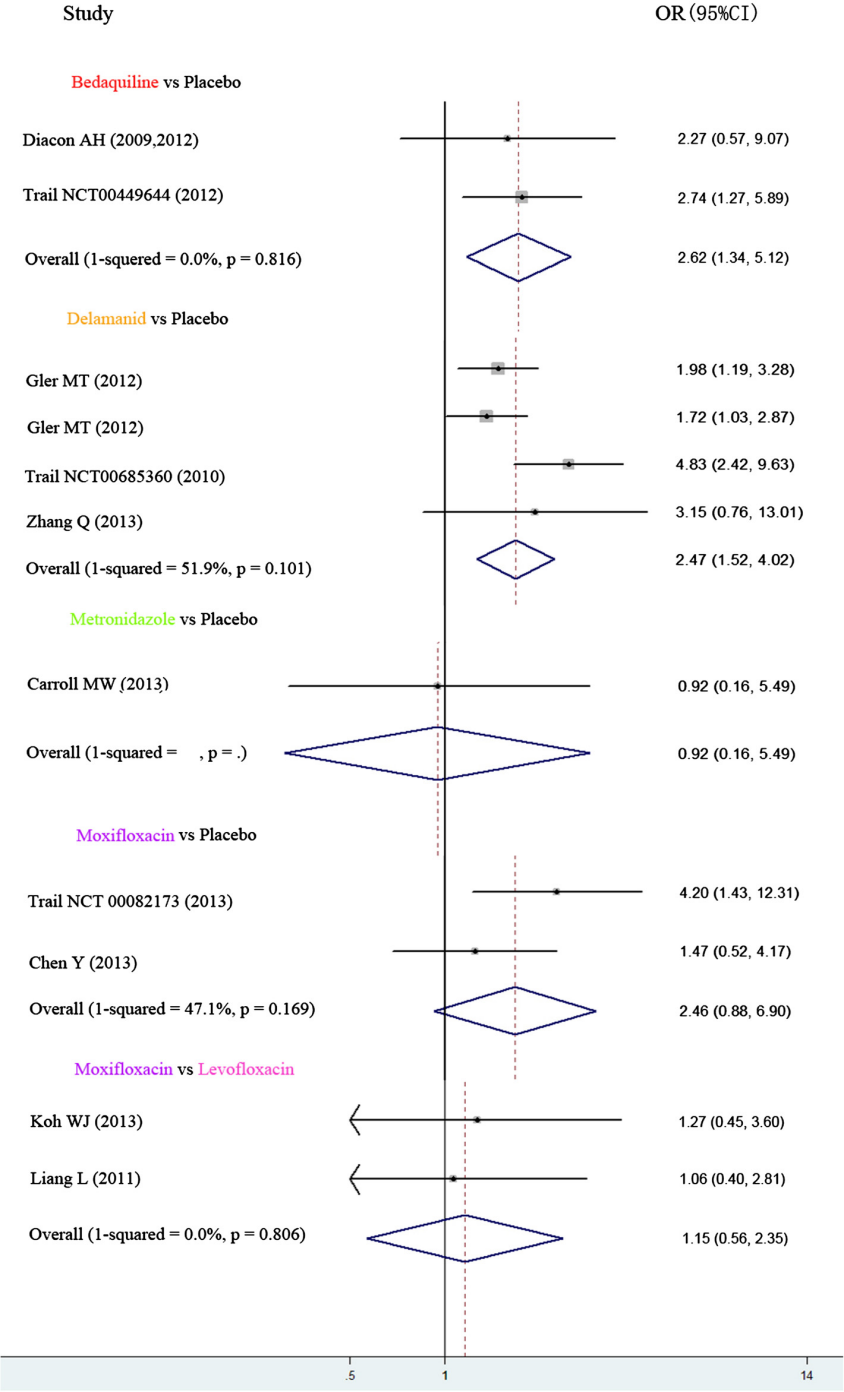
Direct comparisons of efficacy between each pair of antitubercular treatment. The short horizontal represent credibility interval, the cube located in the middle of the horizontal represent odds ratio. The midcourt line represent OR = 1. The rhombus in the final of each studies indicated aggregated results of all the clinical trials. The short horizontal or rhombus was intersected with midcourt line means no significant difference. The short horizontal or rhombus was on the midcourt line’s left means the placebo was more effective.

**Figure 4:**
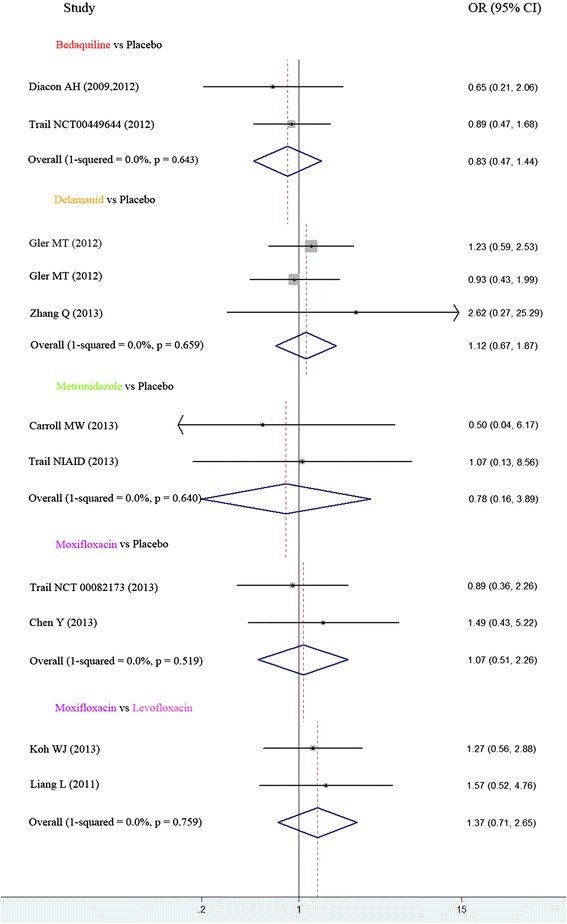
Direct comparisons of acceptability between each pair of antitubercular treatment. The short horizontal represent credibility interval, the cube located in the middle of the horizontal represent odds ratio. The midcourt line represent OR = 1. The rhombus in the final of each studies indicated aggregated results of all the clinical trials. The short horizontal or rhombus was intersected with midcourt line means no significant difference. The short horizontal or rhombus was on the midcourt line’s left means the placebo was more acceptable.

We extracted detailed data from eleven clinical trials to perform analysis of efficacy and acceptability of five antitubercular drugs. The possible pair wise comparisons between bedaquiline, delamanid, levofloxacin, metronidazole, moxifloxacin and placebo had been studied directly in one or more trials. Figure 3 and 4 shows the odds ratios for each of these direct comparisons. The direct comparisons (Figure 3) showed that in terms of efficacy, results derived from these drug comparisons showed no statistical significance at the 95% confidential interval if the pooled OR included 1. For acceptability (Figure 4), none of the comparisons had significance.

Overall, heterogeneity was moderate, although for some comparisons the 95% CI included values that showed low, moderate or no heterogeneity, reflecting the small number of included studies for each pair-wise comparison. In the meta-analyses of direct comparisons of efficacy, we found that 2 values were a little higher for the comparisons delamanid vs. placebo and moxifloxacin vs. placebo, (I^2^ = 51.9% and 47.1% respectively). There was no heterogeneity between the two trials of bedaquiline vs. placebo (I^2^ = 0.0%) and levofloxacin vs. moxifloxacin (I^2^ = 0.0%). The following one comparisons had only a single trial and therefore heterogeneity could not be evaluated (Figure 3). In the meta-analyses of acceptability, no heterogeneity was found in all the comparisons (Figure 4).

### Indirect comparisons

**Figure 5:**
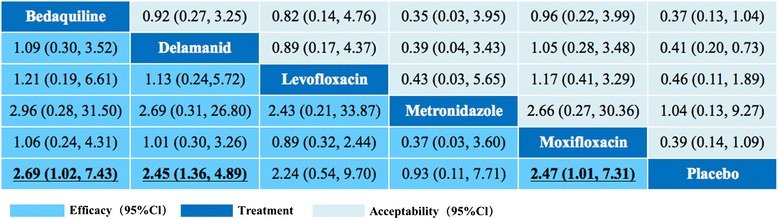
Efficacy and acceptability of the five antituberculosis drugs. Drugs are reported in alphabetical order. Comparisons between drugs should be read from left to right. Results are presented as comparisons of ORs the in the horizontal treatment rows compared with the ORs in the vertical treatment columns. For efficacy, ORs higher than 1 means the upper-left drug was more effective. For acceptability, ORs higher than 1 means the lower-right drug was more acceptable. Significant results are in bold and underscored. OR = Odds ratio. CI = credibility interval.

Figure 5 shows the results of indirect comparisons and the OR was the basis for determining effect size [16,19]. Indirect comparisons could provide pair-wise comparisons when no clinical randomized controlled trials are available. No statistically significant difference was found between bedaquiline, delamanid, levofloxacin, metronidazole and moxifloxacin. In terms of acceptability, there was no statistically significant difference between each of the compared interventions. We used R2Winbugs to conduct the convergence assessment of model (Additional file 3: Figure S2). The parameter totresdev assessed model fit in our statistic method.

We used placebo and bedaquiline as reference compounds to analyze the efficacy and acceptability of the five antituberculosis drugs. With efficacy of placebo as the standard of comparison, bedaquiline, delamanid and moxifloxacin indicated statistically more effective than placebo (Additional file 4: Figure S3A). With bedaquiline as the standard of comparison, there have no significant difference between these five antituberculosis drugs (Additional file 5: Figure S4A). With acceptability of placebo and bedaquiline as the standard of comparison, there was no obvious difference between the interventions (Additional file 4: Figure S3B and Additional file 5: Figure S4B).

## Discussion

Our analysis was based on eleven clinical trials that included 1472 individuals who had been randomly assigned to five antituberculosis drugs used to treat MDR-TB. Our findings might help to choose the most efficacious and acceptable drug among the antituberculosis drugs used for treatment of MDR-TB. Some antituberculosis drugs showed differences in terms of statistical and clinical significance. In terms of response, there was no statistically significant difference in terms of the efficacy and acceptability among these five antituberculosis drugs.

Levofloxacin and moxifloxacin which belongs to fluoroquinolones are the only two drugs that have been directly compared (apart from placebo) among the five antituberculosis drugs. Based on a retrospective analysis, Lee J and co-workers [20] reported that both levofloxacin and moxifloxacin showed equivalent efficacy for treating MDR-TB. Furthermore, Jiang RH et al, indicated that levofloxacin was similar to moxifloxacin in terms of efficacy when used for the treatment of MDR-TB [21]. In the current study, we also found that there was no significant difference between levofloxacin and moxifloxacin in treating MDR-TB (Figure 5).

Inevitably, several limitations are associated with our study. First, most of the antituberculosis drugs for MDR-TB were still in Phase I, II or III clinical trials and therefore the number of eligible articles was limited. The sample size of studies was generally small and most of them comprised of less than 50 patients (Table 1). Although the heterogeneity of comparisons in some trials appeared to be moderate, sample size was not large enough to make any conclusions concerning the equivalence of these effects. Second, for the eligible records, most clinical trials compared drugs with placebo, and those which made direct comparisons between the five antituberculosis drugs was very limited (Figure 2).Third, many studies dealt with a heterogeneous population of MDR-TB patients who were resistant to different antibiotics and therefore this may have an impact on the result. Fourth, the duration treatment and dose of drug varied between the studies. The duration of the treatment course was in the range of 8 to 72 weeks (Table 1). Finally, we chose 8-24 weeks as the time period during which sputum culture-conversion was assessed. However, it is possible that some patients may still be receiving treatment during this time. For some trials, the result was displayed in mean times of sputum culture-conversion, and it may show no difference between drug and placebo groups at 8-24 weeks.

## Conclusion

There is insufficient evidence to suggest that any one of the five antitubercular drugs (bedaquiline, delamanid, levofloxacin, metronidazole and moxifloxacin) has superior efficacy compared to the others. Further randomized controlled trials are needed to verify and confirm this conclusion. In the future, our research will focus on the emergence of new studies, especially large-sample studies.

## Additional files

Additional file 1: Table S1. New potential antimicrobial agents for the treatment of MDR-TB. Additional file 2: Figure S1. Risk of bias graph of the study. Additional file 3: Figure S2. Convergence of the model, totresdev and deviance of network meta- analysis. Additional file 4: Figure S3. Efficacy and acceptability using placebo as reference compound. A: Efficacy using placebo as reference compound; B: Acceptability using placebo as reference compound. Additional file 5: Figure S4. Efficacy and acceptability using bedaquiline as reference compound. A: Efficacy using bedaquiline as reference compound; B: Acceptability using bedaquiline as reference compound.

## Abbreviations

TB: Tuberculosis
MDR-TB: Multidrug resistance tuberculosis
XDR-TB: Extensively drug tuberculosis resistant
INH: Isoniazid
RMP: Rifampin
WHO: World Health Organization
ORs: Odds ratios
FDA: Food and Drug Administration
PMDA: Pharmaceutical and Medical Devices Agency
