# Supplementary material for: Comparative efficacy and acceptability of five anti-tubercular drugs in treatment of multidrug resistant tuberculosis: a network meta-analysis
Source: J Clin Bioinforma. 2015 Apr 28;5:5. doi: 10.1186/s13336-015-0020-x (PMC4416256; doi:10.1186/s13336-015-0020-x)
Supplement: Additional file 1: Table S1. — New potential antimicrobial agents for the treatment of MDR-TB. [file 13336_2015_20_MOESM1_ESM.docx]

**Table S1**

**Table S1. New potential antimicrobial agents for the treatment of MDR-TB**

| **Class** | **Drug** | **Stage of drug development** |
| --- | --- | --- |
| Diaryquinoline | Bedaquiline (TMC-207) | Phase III trials |
| Ethylene diamine | SQ 109 | Phase II trials |
| Fluoroquinolone | Gatifloxacin | Phase III trials |
|  | Levofloxacin | Phase III trials |
|  | Moxifloxacin | Phase III trials |
| Nitroimidazole | Delamanid (OPC-67683) | Phase III trials |
|  | Metronidazole | Phase II trials |
|  | PA-824 | Phase II trials |
| Oxazolidinone | AZD-5847 | Phase I trials |
|  | Linezolid | Phase II trials |
|  | Sutezolid (PNU-100480) | Phase II trials |
| Pyrrole | Sudoterb(LL3858) | Phase I trials |
| Riminophenazine | Clofazimine | Phase II trials |
