# Supplementary figures and images for: Comparative efficacy and acceptability of five anti-tubercular drugs in treatment of multidrug resistant tuberculosis: a network meta-analysis
Source: J Clin Bioinforma. 2015 Apr 28;5:5. doi: 10.1186/s13336-015-0020-x (PMC4416256; doi:10.1186/s13336-015-0020-x)

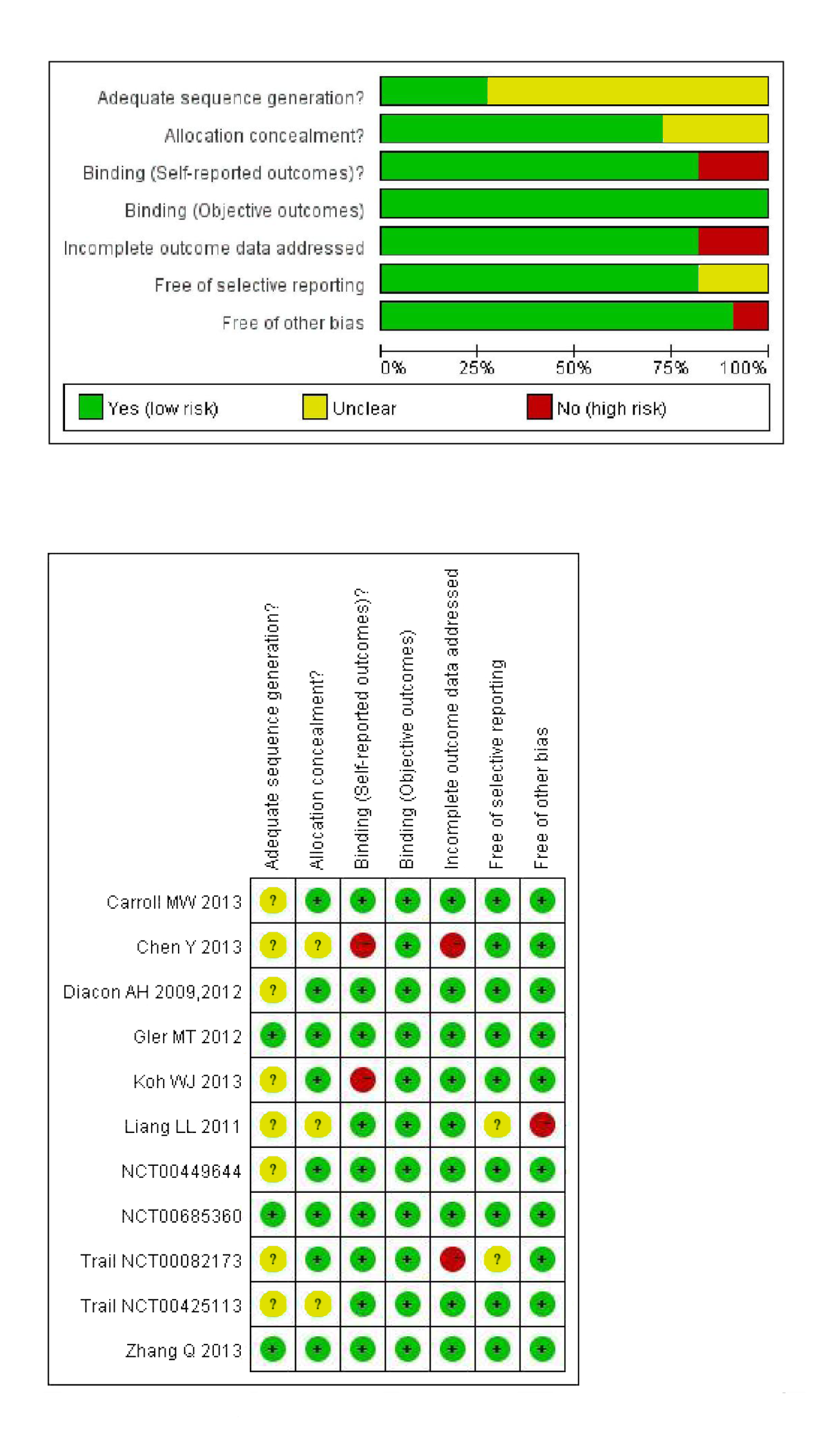

Supplement: Additional file 2: Figure S1. — Risk of bias graph of the study. [file 13336_2015_20_MOESM2_ESM.tiff]

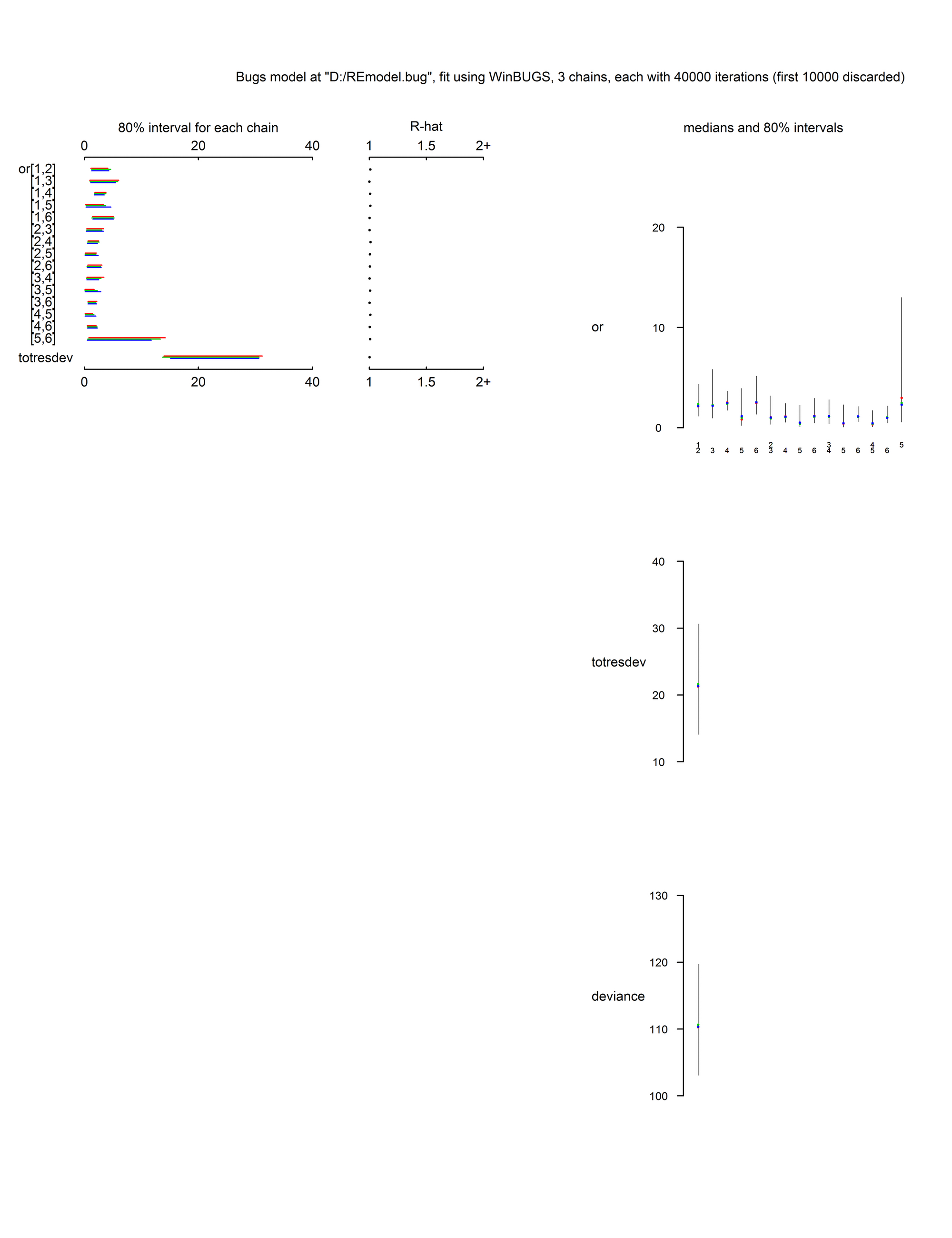

Supplement: Additional file 3: Figure S2. — Convergence of the model, totresdev and deviance of network meta- analysis. [file 13336_2015_20_MOESM3_ESM.tiff]

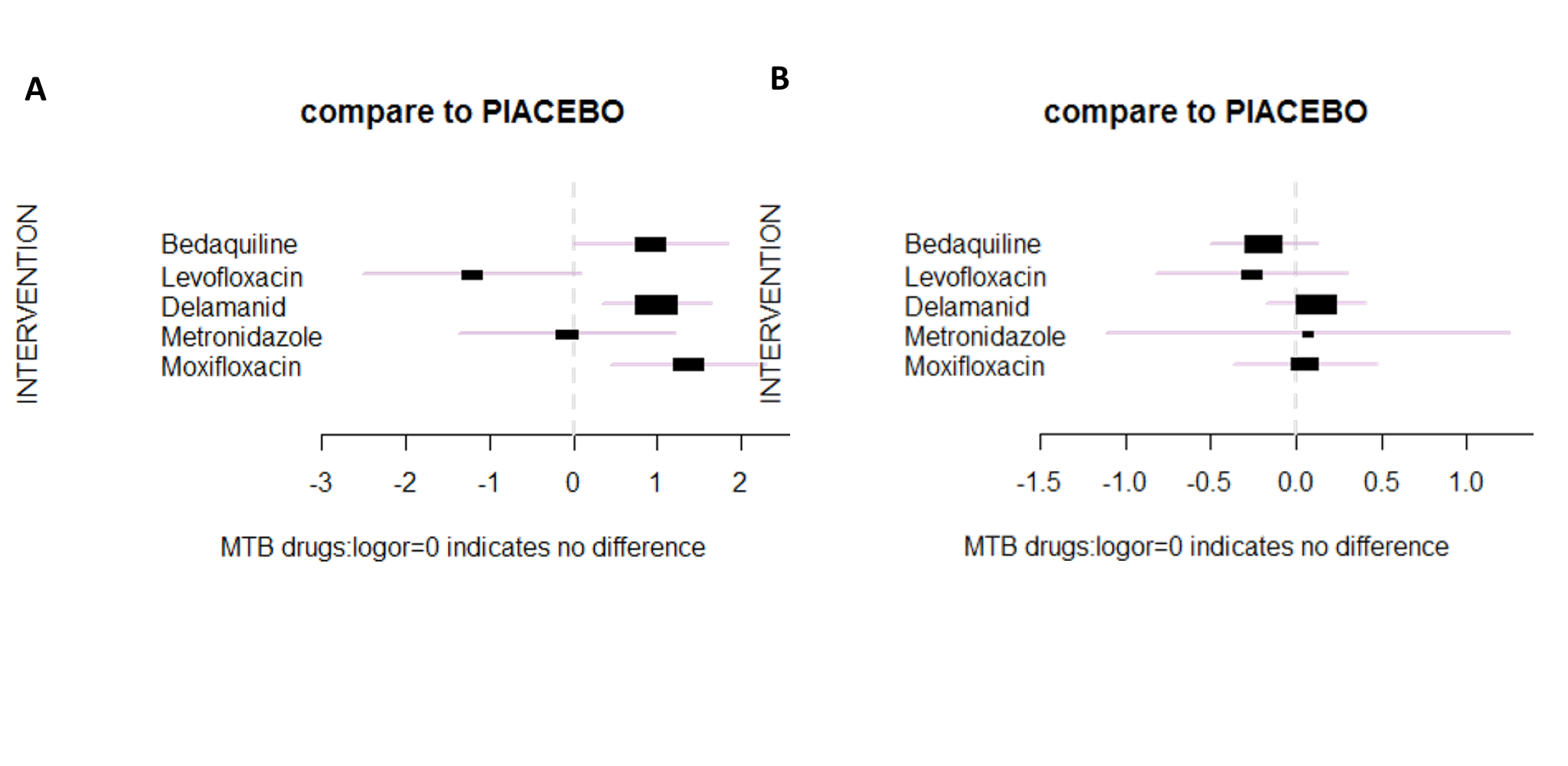

Supplement: Additional file 4: Figure S3. — Efficacy and acceptability using placebo as reference compound. A: Efficacy using placebo as reference compound; B: Acceptability using placebo as reference compound. [file 13336_2015_20_MOESM4_ESM.tiff]

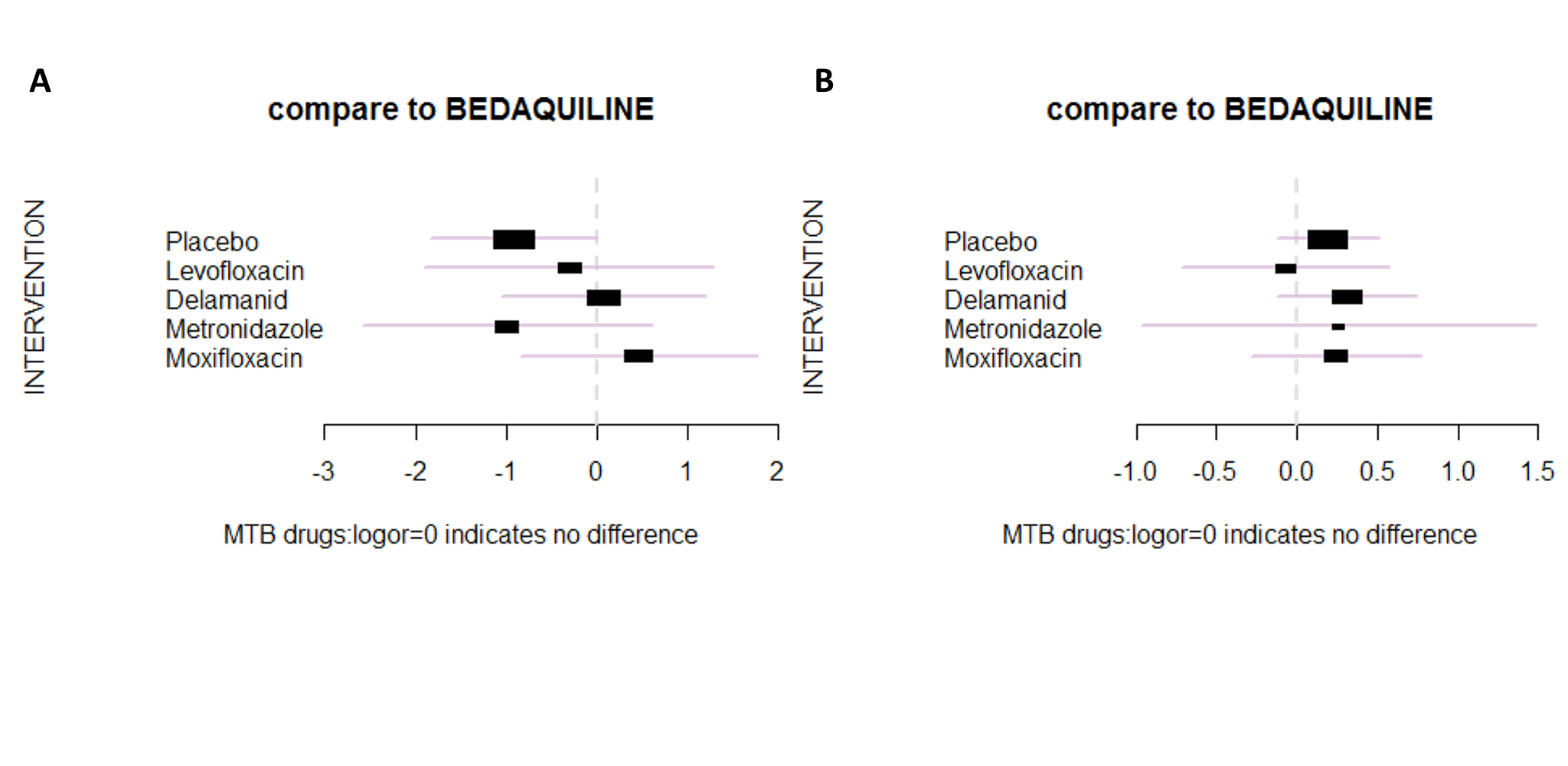

Supplement: Additional file 5: Figure S4. — Efficacy and acceptability using bedaquiline as reference compound. A: Efficacy using bedaquiline as reference compound; B: Acceptability using bedaquiline as reference compound. [file 13336_2015_20_MOESM5_ESM.tiff]
